# PReS-FINAL-2363: Behçet's disease in children: the Great Ormond Street Hospital experience

**DOI:** 10.1186/1546-0096-11-S2-P353

**Published:** 2013-12-05

**Authors:** S Nanthapisal, D Eleftheriou, Y Hong, N Klein, PA Brogan

**Affiliations:** 1 UCL Institute of Child Health, London, UK; 2 Paediatrics, Thammasat University, Pathumthani, Thailand

## Introduction

Behçet's disease (BD) is rare in childhood and remains challenging in diagnosis and lack of evidence-based data for its treatment. Hence there is an urgent need to understand the scope of the disease in children.

## Objectives

The aim of this study is to describe the clinical spectrum and the therapies used to treat children with Behçet's disease (BD) in children.

## Methods

46 patients (22 male) were identified with a positive family history of BD in 6 cases. Age of onset was 4.87 (0.04-15.71) years with a time to diagnosis of 3.74 (0.25-13.48) years. The main clinical features at presentation were: recurrent oral ulceration (87%), genital ulceration (20%), cutaneous symptoms (11%), fever (30%), gastrointestinal symptoms (26%), musculoskeletal (22%). uveitis (2%). Recurrent genital ulceration was significantly more common in female patients (*P *= 0.044). The majority of children were treated with colchicine (74%) and corticosteroid (41%). Anti TNF-a treatment was reserved for severe and/or refractory cases (15%). There was a median of 2 (range 0-12) episodes of oral ulceration per year after the treatment. Interestingly only 10 patients fulfilled The International Study Group (ISG) BD diagnostic criteria.

## Results

46 patients (22 male) were identified with a positive family history of BD in 6 cases. Age of onset was 4.87 (0.04-15.71) years with a time to diagnosis of 3.74 (0.25-13.48) years. The main clinical features at presentation were: recurrent oral ulceration (87%), genital ulceration (20%), cutaneous symptoms (11%), fever (30%), gastrointestinal symptoms (26%), musculoskeletal (22%). uveitis (2%). Recurrent genital ulceration was significantly more common in female patients (*P *= 0.044). The majority of children were treated with colchicine (74%) and corticosteroid (41%). Anti TNF-a treatment was reserved for severe and/or refractory cases (15%). There was a median of 2 (range 0-12) episodes of oral ulceration per year after the treatment. Interestingly only 10 patients fulfilled The International Study Group (ISG) BD diagnostic criteria.

## Conclusion

Although most cases were diagnosed in late childhood the first presentation was as early as 1 month old. Delay in diagnosis due to incomplete presentation in certain cases. Oral ulceration was the most common presenting symptom. Uveitis was less frequent than previous series. A range of drugs was used including biologic therapy for severe cases.

## Disclosure of interest

None declared.

